# Stress in Regulation of GABA Amygdala System and Relevance to Neuropsychiatric Diseases

**DOI:** 10.3389/fnins.2018.00562

**Published:** 2018-08-14

**Authors:** Fan Jie, Guanghao Yin, Wei Yang, Modi Yang, Shuohui Gao, Jiayin Lv, Bingjin Li

**Affiliations:** ^1^Jilin Provincial Key Laboratory on Molecular and Chemical Genetic, The Second Hospital of Jilin University, Changchun, China; ^2^Department of Gastrointestinal Colorectal Surgery, China-Japan Union Hospital of Jilin University, Changchun, China; ^3^Department of Orthopedics, China-Japan Union Hospital of Jilin University, Changchun, China

**Keywords:** amygdala, GABAergic, chronic stress, acute stress, anxiety

## Abstract

The amygdala is an almond-shaped nucleus located deep and medially within the temporal lobe and is thought to play a crucial role in the regulation of emotional processes. GABAergic neurotransmission inhibits the amygdala and prevents us from generating inappropriate emotional and behavioral responses. Stress may cause the reduction of the GABAergic interneuronal network and the development of neuropsychological diseases. In this review, we summarize the recent evidence investigating the possible mechanisms underlying GABAergic control of the amygdala and its interaction with acute and chronic stress. Taken together, this study may contribute to future progress in finding new approaches to reverse the attenuation of GABAergic neurotransmission induced by stress in the amygdala.

## Introduction

The amygdala is an almond-shaped structure located within the temporal lobes of the brain and it plays a key role in the processing of fearful and unpleasant stimuli (Aroniadou-Anderjaska et al., 2007; Bzdok et al., 2013). Previous studies have demonstrated that amygdala nuclei participate in the storage and retrieval of conditioned fear and fear memory (Sah et al., 2003; Herry et al., 2008; Pape and Pare, 2010). The nuclei of the amygdaloid complex can be grouped, according to their embryological origins, into three subdivisions: the centromedial, the cortical, and the basolateral complexes groups (Knapska et al., 2007; Spampanato et al., 2011). These are functionally relevant subdivisions with a little bit of difference. Different nuclei within the amygdala appear to process diverse aspects of stress. The basolateral nucleus (BLA) is a cortical-like structure located in the dorsal amygdala and is involved in the regulation of behavioral and physiological stress responses (Bhatnagar et al., 2004). The central amygdala (CeA) has also been reported to play a crucial role in physiological responses to stressors, such as fearful stimuli, stressful stimuli, and some drug-related stimuli (Gilpin et al., 2015). In addition, accumulating evidence suggests that a key subdivision of the extended amygdala, named the bed nucleus of the stria terminalis (BNST), is involved in anxiety and stress (Li et al., 2012).

The networks of γ-aminobutyric acid-ergic (GABAergic) interneurons in the amygdala are very important components of the brain’s inhibitory circuits (Stefanits et al., 2018). This neurotransmitter is necessary for keeping a balance between neuronal excitation and inhibition (Klausberger and Somogyi, 2008). The BLA contains both glutamatergic principal neurons and GABAergic interneurons (Bhatnagar et al., 2004). The glutamatergic neurons are firmly regulated by a comparatively small population of GABAergic inhibitory neurons (Prager et al., 2016). Destruction of GABAergic inhibition in the BLA can cause behavioral hyperexcitability, such as increased anxiety and depression, emotional dysregulation, and development of seizure activity (Prager et al., 2016). The CeA serves as a major output nucleus of the amygdala by converging inputs from the BLA (Li et al., 2017). In contrast with the BLA, the CeA is only composed of GABAergic neurons (Spampanato et al., 2011). Moreover, the BLA, the CeA, and their connections play a crucial role in the modulation GABAergic control in the amygdala. These amygdala GABAergic neurons are hence adequately positioned to play a central role in the regulation of stress. However, much less is known about the interaction between the amygdala’s GABAergic inhibitory system and stress.

Stress is becoming increasingly inevitable in daily life, causing a series of physiological and behavioral responses that significantly alter emotional and behavioral states (Dallman et al., 2003). The way in which stressors impact emotional states depends on a variety of biological and environmental factors (Ulrich-Lai et al., 2015). There are already some studies in experimental animals which investigated the role of the amygdala’s GABAergic neuronal system in the regulation of stress. For example, Giachero et al. (2013) showed that threatening stress induced attenuation of GABAergic neurotransmission in BLA, and compelling evidence has shown that wistar kyoto rats presented decreased GABAergic activation in the BLA after a 2.0 mA shock (Jiao et al., 2011). A reduction in c-Fos expression in GABAergic interneurons of the BLA was found in postweaning rats who had been subjected to social isolation (Lukkes et al., 2012). Wang GY demonstrated that chronic or acute administration of dexamethasone (DEX) upregulates GABA release and GABAergic neuronal spiking in the amygdala (Wang et al., 2016). Liu ZP demonstrated that both chronic mild stress and unpredictable stress leads to an everlasting loss of tonic GABA_A_ receptor current in the projection neurons of the LA (Liu et al., 2014). These studies indicate the direct relationship between stress and GABA-modulation in the amygdala. We conducted a systematic review combining both preclinical and clinical evidence to evaluate how stress may influence the GABAergic system in the amygdala.

## Function and Structure of GABA and GABA Receptors

GABA is a crucial inhibitory neurotransmitter of the brain and is the primary neurotransmitter of at least one-third of all central nervous system neurons (Bloom and Iversen, 1971). The classes of GABA receptors include GABA_A_, GABA_B_, and GABA_A_-rho (formerly considered GABA_C_) receptors (Olsen and Sieghart, 2008, 2009). GABA_A_ and GABA_C_ belong to the super family of pentameric ligand-gated ion channels (Enz, 2001). The GABA_A_ receptor is composed of five transmembrane protein subunits including two α subunits, two β subunits, and one γ subunit (Sieghart and Sperk, 2002). Heterooligomeric GABA_C_ receptors are composed of three GABA_C_ receptor ρ subunits (ρ1, ρ2, and ρ3) (Enz, 2001). GABA_B_ receptors are made up of GABA_B1_ and GABA_B2_ subunits (Jiang et al., 2012). Ionotropic GABA_A_ and GABA_C_ receptors’ subunits surround a chloride channel. The metabotropic GABA_B_ receptor is coupled to G-proteins and operates by modulating calcium or potassium channels (Enz and Cutting, 1998). GABA_A_ receptors produce a rapid inhibition (Sieghart, 2006), while GABA_B_ receptors are coupled with G-proteins to produce slow and prolonged inhibitory responses (Bowery, 2010). GABA_C_ receptors are more highly localized in axon terminals of bipolar cells compared to GABA_A_ receptors (Enz and Cutting, 1998; McCall et al., 2002). Activation of GABA_A_ receptors can cause a massive increase in chloride conductance through the cell membrane (Mody and Pearce, 2004). Furthermore, low concentrations of GABA can persistently activate extrasynaptic GABA_A_ receptors and cause a sustaining inhibitory state, meaning that the neuron will not present a normal response to excitatory stimuli (Farrant and Nusser, 2005). GABA_B_ receptors play an important role in regulating pre- and postsynapses (Xu et al., 2014). It is recognized that GABA_B_ receptors have an influence on the activity and signaling of glutamate receptors both physiologically and pathologically (Kantamneni, 2015). Some studies have suggested that GABA_C_ receptors are involved in sleep-waking conduction (Arnaud et al., 2001), emotion and memory (Chebib et al., 2009), apoptosis (Yang et al., 2003), and hormone release in the pituitary (Boue-Grabot et al., 2000). Known GABA_C_ receptor binding proteins do not interact with other types of GABA receptors, implying that GABA_C_ receptors have unique pharmacological and physical characteristics (Enz, 2001).

## GABAergic Control of the Amygdala

**FIGURE 1:**
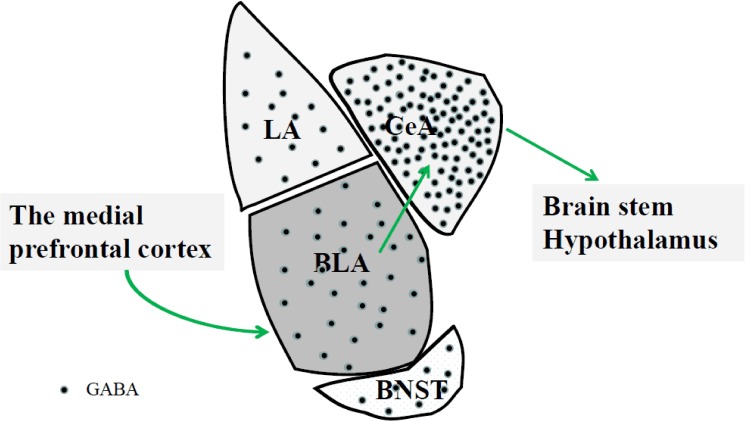
The medial prefrontal cortex can affect BLA GABAergic system. The BLA neurons indirectly project to the CeA via GABAergic interneurons. Projections from CeA mainly target to the hypothalamus and brainstem involved in physiological and behavioral responses to stress. BLA, basolateral nucleus; CeA, central amygdala; BNST, bed nucleus of the stria terminalis; LA, lateral amygdala.

As we mentioned above, the amygdala is composed of a number of distinct nuclei including BLA, lateral amygdala (LA), CeA and a key subdivision of the extended amygdala, BNST. The different distribution of GABA is accompanied by various functions in each sub nuclei of amygdaloid complex. Major afferent signals from the medial prefrontal cortex reach the amygdala mainly via the BLA and LA, while efferent signals tend to originate through the CeA (Etkin, 2010). Inhibitory GABAergic neurons project from the CeA to the hypothalamus and brainstem (Jongen-Rêlo and Amaral, 1998). The amygdala is inhibited by the cortex through the activation of local GABAergic interneurons. In addition, this inhibition is significantly decreased when dopamine is released during heightened emotional states (Mcdonald et al., 1996; Dallman et al., 2003; Pinto and Sesack, 2008). The modulation of emotional responses by the BLA is mainly determined by the balance of excitatory and inhibitory inputs to its dominated neurons which are tightly controlled by GABAergic interneurons (Równiak et al., 2017). As mentioned above, the GABAergic neurons of the amygdala modulate activation of the CeA via projections from the BLA (Nuss, 2015). Optogenetic techniques selectively isolate distinct neural circuits and help us in the identification of novel brain pathways. Sparta et al. reported that photostimulation of BNST efferents resulted in a behavioral phenotype of the anxiety-like state (Sparta et al., 2013). **Figure 1** shows a schematic of the GABA distribution and the projections in each sub nuclei of amygdaloid complex. The GABA synthetic enzyme glutamic acid decarboxylase (GAD) 65 and a second GAD isozyme, GAD67, are important components involved in the activity-dependent modulation of the amygdala’s GABAergic system (Müller et al., 2015). Some animal experiments have indicated that administration of GABA receptor agonists or antagonists into the amygdala can influence the concentration of GABA (Sanders and Shekhar, 1995; Barbalho et al., 2009). These have included GABAergic agonists such as benzodiazepines (Sigel and Lüscher, 2011), Zol (Alò et al., 2015), muscimol (Jasnow and Huhman, 2001; Liu et al., 2009), diazepam, and abecarnil (Eva et al., 2004). GABAergic antagonists have included baclofen (Gorsane et al., 2012), bicuculline (Liu et al., 2009), and FG-7124 (Eva et al., 2004; Lukkes et al., 2012). Benzodiazepines, Zol, diazepam, and abecarnil bind the subunits of GABA_A_ receptors, and this binding increases the probability of ion channel opening in the presence of GABA (Eva et al., 2004; Alò et al., 2015; Fast and McGann, 2017). On the other hand, baclofen competes with GABA for the same sites on GABA_B_ receptors in the amygdala. It has been proposed that these could be helpful in the therapy of stress-induced disorders (Gorsane et al., 2012).

## Types of Stress

Stress is our body’s way of responding to a variety of demands or threats, and affects many bodily functions, such as metabolic, psychological, and behavioral functions. Stress management can be complicated and difficult to understand because it is influenced by many variables, such as the chronicity, predictability and severity of stress (Nalivaiko, 2011; Steptoe and Kivimäki, 2012; Herman, 2013). Different classification methods divide stress into different types. Stress can be roughly divided into social and non-social (physical) stress (Lee et al., 2016). There are already some reports studying different types of non-social stress in rodents, including restraint stress (Lee et al., 2016), forced swim stress (Suarez-Roca et al., 2008), prenatal stress exposure (Lee et al., 2016) and elevated platform stress (Sickmann et al., 2015). Rodent studies on social stress have included social defeat-induced stress (Rutherford et al., 2014), social mixing stress (Jarvis et al., 2006), and social crowdedness stress (Sachser et al., 2011). Some animal studies looking at the influence of predictable and unpredictable stressor stimuli showed that the latter induces more pronounced behavioral and physiological results (Bassett et al., 1973; Magariños and McEwen, 1995; Marin et al., 2007; Flak et al., 2012; Kopp et al., 2013; Smith et al., 2013). However, most studies do not make a clear distinction between predictable and unpredictable stressor stimuli and focus more on the duration of the stimulus. Furthermore, there have been some reports showing that acute and chronic aversive stimuli cause very different responses (Nalivaiko, 2011; Herman, 2013). Based on the above reasons, we have divided stress into two types: acute stress and chronic stress. There have been many studies demonstrating that various types of stress are involved in the control of the amygdala’s GABAergic neurons. These include acute forced swim stress (Bedse et al., 2015), prenatal stress (Ehrlich et al., 2015), chronic unpredictable restraint stress (Ortiz et al., 2015), and posttraumatic stress (Müller et al., 2015). Stress can also derive from withdraw infections of drugs or continuous alcohol abuse (Stephens and Wand, 2012). Agentia administration can influence the hypothalamic-pituitary-adrenal (HPA) axis and we have categorized it as acute or chronic stress depending on its method of administration. Likewise, we categorized chronic ethanol exposure as chronic stress and acute ethanol exposure as acute stress. We will describe these in two categories according to the classification factors mentioned above.

## Influence of the GABAergic System in Amygdala Responses to Stress

Severe acute stress and chronic stress can influence the amygdala’s stress response through three main regulatory systems: the serotonergic system (Hernández et al., 2016), the catecholaminergic system (Wang et al., 2016), and the HPA axis (Wisłowska-Stanek et al., 2013). Here, we highlight the influence of the GABAergic system in amygdala responses to stress. As we described above, the GABAergic responses to stress also involve many subnuclei of the amygdala, such as the BLA, CeA, LA, and BNST (Zuloaga et al., 2012). In addition, stress influence amygdala’s GABAergic transmission in a cell type- and projection- specific way. Accumulating evidence demonstrates that BLA projections to the CeA distinctly alter motivated behavior (Beyeler et al., 2018). Stress induced persistent anxiety via the extra-amygdala septohypothalamic circuit (Anthony et al., 2014). Liu et al. (2018) found that harmine potentiates the GABAergic transmission onto BLA projection neurons. However, only partially understood about the role of neuronal components of these regions in amygdala circuits. For instance, Yu et al. (2017) showed that PKC-δpositive lateral CeA neurons were “fear-on” neurons as they convey aversive unconditioned stimulus signals. Administration of CRF into the BLA induced pronounced increases in cFos-ir in the CaMKII-ir population and altered the activity of GABAergic interneurons (Rostkowski et al., 2013). The relationship between GABA amygdalar system and stress is complex. Depending on the duration of stress, more influence of the GABAergic system in amygdala responses to acute stress and chronic stress will be discussed separately below.

### GABAergic Control of Amygdala Responses to Acute Stress

Restraint and forced swimming are the most common form of acute stress. Tian et al. (2013) found some new targets that reduce the amygdala’s response to these acute stresses. G-protein-coupled receptor 30, one of the estrogen receptors, is a novel membrane receptor that is highly expressed in the BLA. Additionally, G-protein-coupled receptor 30 expression in the amygdala was substantially increased after acute stress and this correlated with anxiety-like behaviors. Moreover, the G-protein-coupled receptor 30 agonist blocked the down-regulation of GABA_A_ receptors (Tian et al., 2013). Feng et al. found another potential therapy for regulate stress is motilin which can weaken anxiety-like behavior in rats after they have been subjected to forced swimming. Whole-cell recordings from amygdala slices revealed that motilin depolarized the interneurons and promoted GABAergic transmission in the BLA (Feng et al., 2013). Bedse et al. (2015) found that pretreatment with the cannabinoid receptor type 1 receptor antagonist rimonabant blocked the effect of the fatty acid amide hydrolase inhibitor (URB597) on GABA release in the BLA of animals subjected to the acute swim stress. In rats subjected to restraint stress, acute application of corticotropin releasing factor significantly increased inhibitory postsynaptic potentials in the CeA (Ciccocioppo et al., 2014).

Neonatal maternal separation stress is instantaneous but induces long-lasting alterations in emotional behaviors. It was reported that adult rats that had experienced neonatal maternal separation presented an increase in the density of arginine-vasopressin innervation in the amygdala. Furthermore, V1a arginine-vasopressin receptor mRNA was only found in GABAergic neurons, demonstrated by complete co-localization of V1a transcripts in CeA neurons expressing GAD transcripts (Hernández et al., 2016). Another study demonstrated that postweaning social isolation decreased c-Fos expression in a subset of GABAergic interneurons in the BLA of adult female rats (Lukkes et al., 2012).

It is worth noting that the HPA axis can mediate the stress response because it is a neuroendocrine system. Some researchers looked at the effects of rapid glucocorticoid-induced acute stress in the rat BLA. Glucocorticoid administrated to amygdala slices produced a rapid, non-reversible suppression of spontaneous GABAergic synaptic currents (Di et al., 2016). The acute administration of glucocorticoid receptor agonist DEX also upregulated GABA release and GABAergic neuronal spiking (Wang et al., 2016).

Acute ethanol consumption increased GABAergic transmission via the mechanisms involved in both presynaptic and postsynaptic functioning (Varodayan et al., 2017). Although, ethanol facilitated GABAergic transmission in the brain, the activation of cannabinoid receptor type 1 inhibited this effect (Di et al., 2016; Varodayan et al., 2017).

### GABAergic Control of Amygdala Responses to Chronic Stress

Water-deprivation is a kind of chronic stress. Water-deprivation for 24 h in rats enhanced anxiety correlative behavior measured with the elevated plus maze test. This effect was reproduced by bilateral micro infusion of arginine-vasopressin into the CeA. Chronic stress induced by either water-deprivation or arginine-vasopressin infusion was reversed by CeA infusion of a V1a antagonist (Hernández et al., 2016).

In another chronic unpredictable stress model, hamsters were casually subjected to one of three pre-prepared stressful circumstances: food or water deprivation, forced swim test, and endurance in a cold room. Injection of the α1 GABA_A_ receptor subunit agonist (Zol) into the CeA changed elevated plus maze performances (Alò et al., 2015). Chronic, unpredictable stress increased the amplitude of evoked induced pluripotent stem cells and the connectivity between corticotropin releasing factor positive neurons in the CeA and BNST (Partridge et al., 2016). In male rats with chronic unpredictable restraint stress, GAD65 expression in the amygdala negatively correlated with radial arm water maze performances on day 1 in rats subjected to unpredictable restraint stress (Ortiz et al., 2015). Importantly, baseline CeA GABAergic responses were elevated in restrained rats compared with unrestrained rats.

Rats subjected to repeated corticosterone administration showed an increase in anxiety-like behavior, examined using the open field test. The behavioral effects caused by corticosterone injections may because of increased expression of c-Fos in the LA and CeA nuclei of the amygdala and decreased GABA_A_ α-2 subunit density in the CeA of these rats (Skórzewska et al., 2015). These findings are consistent with those of another study (Lussier et al., 2013). Liu et al. (2014) suggested that a lasting loss of tonic but not phasic GABA_A_ receptor currents severely contributes to the prolonged amygdala disinhibition observed after chronic stress. Injection of glucocorticoids during early development may lead to long-term variations in brain function and behavior. The glucocorticoid receptor agonist DEX plays a role in emotion. Postnatal DEX administration in animals caused an increase in cleaved caspase-3 and the expression of a GABAergic calcium-binding protein phenotype in the amygdala (Zuloaga et al., 2011). DEX administration mainly caused a decrease in the number of calretinin immunoreactive cells in the LA of adult female offspring, but no differences were observed in the BLA (Zuloaga et al., 2012). The chronic administration of DEX upregulated GABA release and GABAergic neuronal intensification, and also enhanced the responsiveness of GABA receptors (Wang et al., 2016). Peroxisome proliferator-activated receptors are members of the nuclear hormone receptor family. Peroxisome proliferator-activated receptor agonists such as fenofibrate and tesaglitazar, when administered to mice subjected to a free access two-bottle choice drinking paradigm, provoked a strong brain neuronal signature and targeted a small group of GABAergic interneurons in the amygdala (Ferguson et al., 2014).

Alcohol can cause the dysfunction of the cannabinoid receptor type 1 in many ways. However, a study showed that chronic alcohol exposure disrupted the cannabinoid receptor type 1-associated modulation of the GABAergic system in the rat basolateral amygdala (Varodayan et al., 2017).

Commonly, the prenatal period, infancy, pubescence, and adolescence are critical periods in which animals are more sensitive to stressors than usual (Charmandari et al., 2003, 2012). Ehrlich et al. used a prenatal stress model of maternal depression to test the changes of GABAergic neurotransmission in the amygdala. They found that rats exposed to this stress *in utero* had increased anxiety-like behavior in adulthood. Exposure to prenatal stress also deeply influenced the expression of the chloride transporters K-Cl cotransporter 2 and Na-K-Cl cotransporter 1 in the amygdala, indicating that stress regulates GABAergic function (Ehrlich et al., 2015). Tzanoulinou et al. showed that peripuberty stress may cause a decrease in the expression of GAD and GABA_A_ receptor subunits in all amygdala nuclei present in adult rats (Tzanoulinou et al., 2014). During the juvenile period, rats are particularly vulnerable to stressors. Animals were subjected to a juvenile variable stressor regimen at 27–29 postnatal days (PND), including PND-acute swim stress, PND-elevated platform stress, and PND-restraint stress. The stress-induced regulation of the GABA_A_ receptor subunits was specifically evident in the amygdala (Jacobson-Pick and Richter-Levin, 2012).

**Table 1** summarizes some of the interaction between GABAergic transmission in particular regions of the amygdala and particular types of stress. BLA and CeA seems to be the most relevant regions of GABAergic neurotransmission in the amygdala.

**Table 1:** Influence of the GABAergic system in amygdala responses to stress.

Types of stress		Regions	Changes in GABAergic system	References
Acute	Forced swimming	BLA	Activation of GPR30 increased the inhibitory synaptic transmission	Tian et al., 2013
		BLA	Promote GABAergic transmission	Feng et al., 2013
		BLA	Disrupted the cannabinoid receptor type 1-associated modulation of the GABAergic system	Bedse et al., 2015
	Restraint stress	CeA	Elevate baseline GABAergic responses	Ciccocioppo et al., 2014
	Maternal separation	CeA	Increase in the density V1a transcripts of GABAergic neurons	Hernández et al., 2016
		BLA	Decrease c-Fos expression in a subset of GABAergic interneurons	Lukkes et al., 2012
	Glucocorticoid administration	BLA	Suppress spontaneous GABAergic synaptic currents	Di et al., 2016
	DEX administration		Upregulate GABA release and GABAergic neuronal spiking	Wang et al., 2016
	Ethanol consumption	BLA	Increase GABAergic transmission	Varodayan et al., 2017
Chronic	Water-deprivation	CeA	Increase in the density V1a transcripts of GABAergic neurons	Hernández et al., 2016
	Chronic unpredictable	CeA	Regulation of GABAA receptors	Alò et al., 2015
		CeA and BNST	Promote GABAergic transmission	Partridge et al., 2016
		CeA	Down-regulation of GAD65 expression	Ortiz et al., 2015
	Corticosterone administration	LA and CeA	Decreased GABAA α-2 subunit density	Skórzewska et al., 2015
	DEX administration		Increase cleaved caspase-3 and GABAergic calcium-binding protein	Zuloaga et al., 2011
			Enhanced the responsiveness of GABA receptors	Wang et al., 2016
	Ethanol consumption	BLA	Disrupted the cannabinoid receptor type 1-associated modulation of the GABAergic system	Varodayan et al., 2017
	Prenatal stress		Influenced the chloride transporters K-Cl cotransporter 2, Na-K-Cl cotransporter 1	Ehrlich et al., 2015
	Peripuberty stress	LA, BLA, and CeA	Decrease in the expression of GAD and GABAA receptor subunits	Tzanoulinou et al., 2014
			Regulation of the GABAA receptor subunits	Jacobson-Pick and Richter-Levin, 2012


BLA, basolateral nucleus; CeA, central amygdala; BNST, bed nucleus of the stria terminalis; LA, lateral amygdala; GPR30, oxG-protein-coupled receptor 30;GABA, oxγ-aminobutyric acid; BLA, basolateral nucleus; GAD, glutamic acid decarboxylase.

## GABAergic Control of the Amygdala and Relevance to Neuropsychiatric Diseases

There may be an interaction between stress and neuropsychiatric diseases. A number of studies have demonstrated this interaction (Schneiderman et al., 2005). Animal studies showed that exposure to acute or chronic stress can induce morphological and functional changes in amygdala. These changes in amygdala can cause individual susceptibility to anxiety disorders (Sandi and Richter-Levin, 2009). Chronic stress generally cause the development of psychological problems such as delusions (Kingston and Schuurmans-Stekhoven, 2016), depression (Norman and Malla, 1993; Corcoran et al., 2003; Hammen, 2005), and anxiety (Schlotz et al., 2011; Saveanu and Nemeroff, 2012). There are also reports that chronic stress is possibly a major cause of depression, and that acute severe stress leads to anxiety (Wang et al., 2016). Khansari et al. demonstrated that chronic stress is linked to Alzheimer’s disease (Khansari et al., 1990). In fact, GABAergic control of the amygdala is mostly relevant to anxiety. Some animal researches have shown that administration of corticosterone into the CeA can induce anxiety-like behavior. This animal model imitate the depressed effect of chronic stress on GABAergic tonic inhibition in LA (Myers et al., 2005). There are also some studies that show an association between the amygdala’s GABA interneuronal network and alcohol addiction (Nie et al., 2004, 2009; Bajo et al., 2008). Aroniadou et al. reported a key role of the amygdala’s GABAergic control in epilepsy. The stress-induced damage to the noradrenergic system, promoting GABA release in the BLA, may underlie the stress-induced exacerbation of seizure activity in epileptic patients (Aroniadou-Anderjaska et al., 2007). However, more recent studies have suggested that stress itself does not enhance the risk of developing a disorder, but that it is the perception that stress affects health that is destructive (Keller et al., 2012). For instance, when humans are exposed to chronic stress, steady changes in their physiological and emotional state are the most involved in changes that could lead to illness (Tsoory et al., 2007; Jeronimus et al., 2014). More clinical evidence is needed to better understand stress and to be able to attenuate the effects of stress. This study provides a novel understanding of the interaction between GABAergic transmission in particular regions of the amygdala and particular types of stress.

## Author Contributions

FJ, GY, WY, SG, JL, and BL participated in the discussion of the paper. SG, JL, and BL provided the critical revisions. All authors approved the final version of the manuscript for submission.

## Conflict of Interest Statement

The authors declare that the research was conducted in the absence of any commercial or financial relationships that could be construed as a potential conflict of interest.

## Abbreviations

BLA: basolateral nucleus
BNST: stria terminalis
CeA: central amygdala
DEX: dexamethasone
GABA: γ-aminobutyric acid
GAD: glutamic acid decarboxylase
HPA: hypothalamic-pituitary-adrenal
LA: lateral amygdala
PND: postnatal days
